# The iFat1 transgene permits conditional endogenous n-3 PUFA enrichment both in vitro and in vivo

**DOI:** 10.1007/s11248-014-9788-x

**Published:** 2014-03-13

**Authors:** Shannon E. Clarke, Jing X. Kang, David W. L. Ma

**Affiliations:** 1 Department of Human Health and Nutritional Sciences, University of Guelph, 50 Stone Road East, Guelph, ON N1G 2W1 Canada; 2 Department of Medicine, Massachusetts General Hospital/Harvard Medical School, Boston, MA 02129 USA

**Keywords:** *fat*-*1*, n-3 Desaturase, Cre-recombinase, loxP

## Abstract

Fat-1 transgenic mice, which endogenously convert n-6 PUFA to n-3 PUFA, are a useful tool in health research; however with this model timing of n-3 PUFA enrichment cannot be directly controlled. To add such capability, the novel Cre-recombinase inducible fat-1 (iFat1) transgenic mouse has been developed. The aim of this study was to characterize the utility of the iFat1 transgene as a model of Cre-inducible endogenous n-3 PUFA enrichment. Functionality of the iFat1 transgene was screened both in vitro and in vivo. In the presence of Cre, the iFat1 transgene resulted in a balancing (*p* < 0.01) of the n-6/n-3 PUFA ratio within phospholipids in the human embryonic kidney 293T cell line. For in vivo analysis, iFat1 transgenic mice were crossed with the R26-Cre-ER^T2^ (Tam-Cre) mouse line, a tamoxifen inducible Cre-expression model. Tam-Cre/iFat1 double hybrids were transiently treated with tamoxifen at 6–7 weeks, then terminated 3 weeks later. Tamoxifen treated mice had increased (*p* < 0.05) tissue n-3 PUFA and ≥two-fold reduction (*p* < 0.05) in the n-6/n-3 PUFA ratio of liver, kidney and muscle phospholipids relative to vehicle treated controls. Collectively these findings suggest that the iFat1 transgenic mouse may be a promising tool to help elucidate the temporal effects through which n-3 PUFA impacts health related outcomes.

## Introduction

In recent years, dietary n-3 PUFA, particularly eicosapentaenoic acid (EPA) and docosahexaenoic acid (DHA), have garnered strong research and public interest due to their numerous reported beneficial health effects (Anderson and Ma 2009; Fetterman and Zdanowicz 2009). However, there remain many fundamental questions regarding their biological role and mechanism of action. In particular, timing of exposure during the lifecycle is becoming increasingly salient as a growing body of research suggests that the origins of chronic disease begins in the early years of life (Calkins and Devaskar 2011). No experimental model currently exists which enables the precise study of timing of exposure of n-3 PUFA. Thus, the objective of this study was to develop an inducible transgenic mouse model for the synthesis of n-3 PUFA.

In 2004, Kang and colleagues described the development of the first transgenic mouse model capable of endogenous n-3 PUFA biosynthesis (Kang et al. 2004). Fat-1 transgenic mice are uniquely able to convert n-6 PUFA to n-3 PUFA through constitutive expression of the *fat*-*1* transgene, an n-3 desaturase native to the roundworm *Caenorhabditis elegans* (*C. elegans*). By contrast, mammals lack this gene and are thus unable to synthesize n-3 PUFA and must be obtained through the diet. To date, this transgenic mouse model has greatly enhanced our insight into the protective effects of lifelong endogenous tissue n-3 PUFA enrichment on numerous health related outcomes including, cancer chemoprevention, bone development, inflammatory/immune pathology, and neurological disease (Kang 2007; Lau et al. 2009a, b; Lebbadi et al. 2011; MacLennan et al. 2013). However, while this well-controlled model is able to minimize diet-based variability and confounding that is inherent to traditional diet feeding approaches, n-3 PUFA enrichment encompasses the full duration of the lifecycle, including critical windows of developmental programming which are suggested to be highly sensitive to n-3 PUFA (Calder et al. 2010; Hilakivi-Clarke 2007; Luijten et al. 2013). As such, the ability to more clearly define critical periods of development during which n-3 PUFA can elicit health related benefits will greatly assist in refining the therapeutic applicability of this class of fatty acids.

The bacteriophage P1 derived Cre-loxP recombinase system is a commonly employed approach to achieve conditional mutagenesis within mammalian cells (Nagy 2000). Through selective inclusion of a loxP flanked regulatory STOP element between a transgene and its driving promoter, Cre-inducible control over transgene activation can be successfully achieved (Lakso et al. 1992). Moreover, since the Cre-loxP recombination system require, at minimum, two independent genetic components to produce conditionality, floxed transgenic mice harbour a more versatile research potential than that which is achievable with more classical transgenic approaches. While alternative murine models with both broad (Wei et al. 2010) and tissue specific (Ji et al. 2009; Kao et al. 2006) patterns of heterologous *C. elegans fat*-*1* gene expression have been reported, a model capable of Cre-inducible *fat*-*1* expression has yet to be described. To this end, we report the development and characterization of a Cre-inducible fat-1 (iFat1) mouse model.

**Fig. 1:**
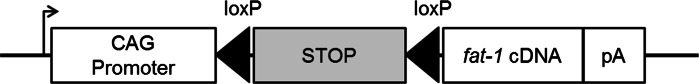
Schematic representation of the iFat1 transgene. The iFat1 transgenic construct consists of a loxP flanked STOP cassette positioned between the codon optimized *fat*-*1* coding cDNA and upstream ubiquitous CAG promoter. This transcriptional regulatory mechanism, with back-up translational block, has been demonstrated to prevent functional activation of downstream transgenes efficiency in the absence of Cre recombinase

The iFat1 transgene consists of a loxP flanked STOP element positioned between the *fat*-*1* coding DNA sequence and its upstream CAG promoter, a chicken β-actin/CMV immediate early enhancer fusion promoter which has been demonstrated to drive a strong ubiquitous pattern of transgene expression in vivo (Okabe et al. 1997; Fig. 1). To protect against uncontrolled transgene activation, the loxP flanked regulatory STOP sequence of the iFat1 transgene is equipped with a C-terminal portion of the yeast *HIS3* gene, an SV40 polyadenylation signal, false translational initiation codon 5′ splice donor site. This transcriptional/translational block, has been demonstrated to prevent functional activation of downstream transgenes with high efficiency in the absence of Cre recombinase (Lakso et al. 1992). This study was designed to evaluate the utility the iFat1 transgene as a model of Cre-inducible *fat*-*1* expression. Function of the iFat1 transgene was screened in vitro using co-transfection experiments in the HEK 293T cell line. For in vivo characterization the Tam-Cre mouse line was selected (Ventura et al. 2007). Tam-Cre mice ubiquitously express a human estrogen receptor-Cre fusion protein which is reliant on administration of the drug tamoxifen for nuclear translocation and subsequent Cre-mediated recombination (Ventura et al. 2007). Using these complementary in vitro and in vivo methodologies we describe, for the first time, a novel transgenic approach for Cre-inducible endogenous n-3 PUFA enrichment.

## Materials and methods

### Transgenic construct and iFat1 model development

The *iFat1* transgenic construct and mouse model were commercially generated (GenOway, Lyon, France). The *fat*-*1* cDNA, codon optimized for efficient mammalian expression were provided by Dr. Jing Kang (Massachusetts General Hospital/Harvard Medical School).

**Fig. 2:**
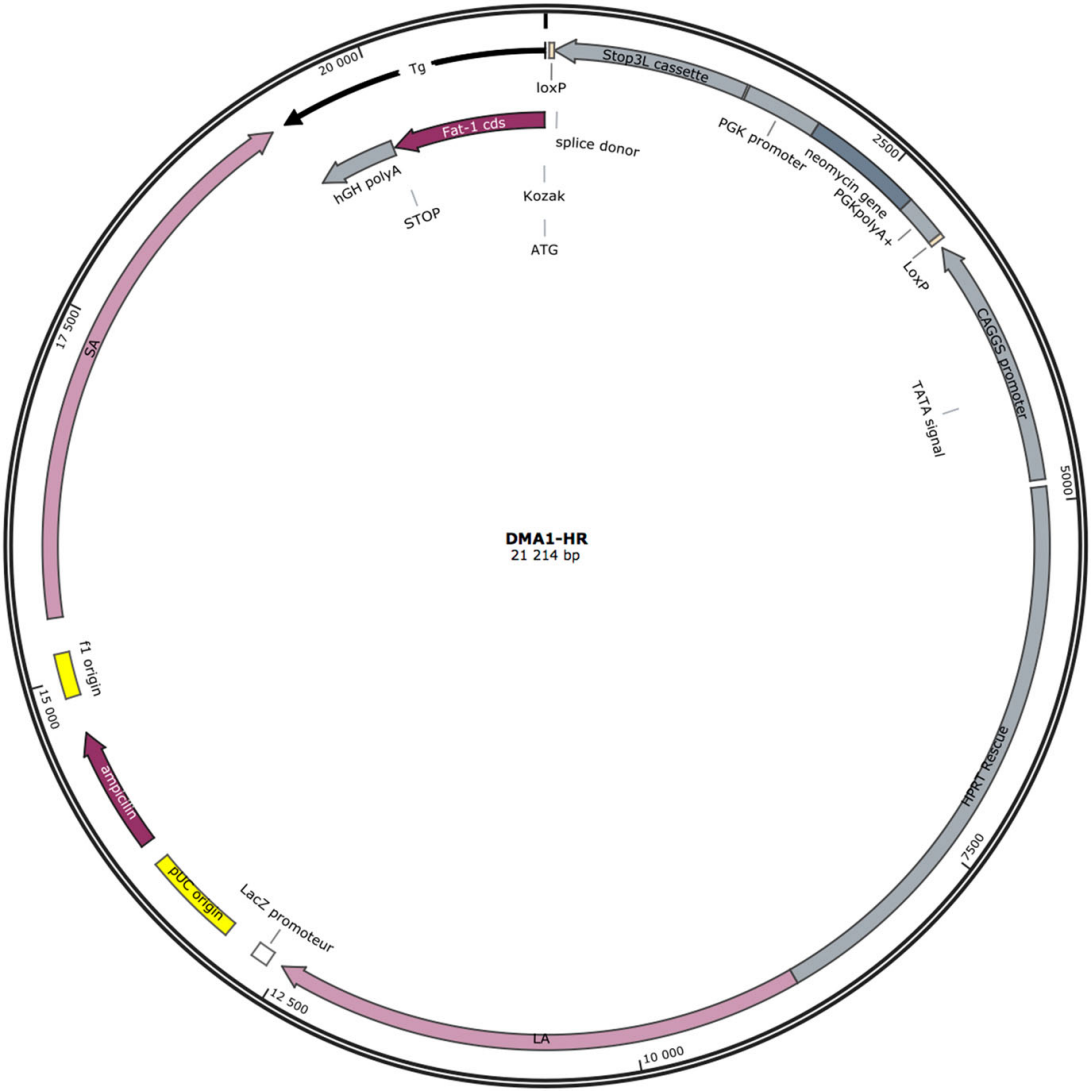
Construct map of DMA1-HR the iFat-1targeting vector (as provided by manufacturer, GenOway). A single copy of the iFat-1 transgene was specifically targeted to the Hprt locus of the X-chromosome using GenOway’s validated Quick Knock-in™ approach

A validated Quick Knock-in™ approach was used to introduce a single copy of the iFat1 transgenic cassette into the hypoxanthine phosphoribosyltransferase (*Hprt*) locus of the X-chromosome through homologous recombination in E14Tg2a embryonic stem cells, a derivative of the 129P2/OlaHsd strain (Fig. 2). The *Hprt* gene codes for a house keeping protein integral to the Salvage Pathway of nucleotide synthesis, an enzymatic cascade which is reliant on the recycled degradation products of nucleotide metabolism as substrate in the synthesis of purine nucleotides. In E14Tg2a embryonic stem cells a 35 kb portion of the *Hprt* locus encompassing the promoter and first two exons has been deleted rendering this cell line solely dependent on the de novo pathway of nucleotide synthesis for survival (Hooper et al. 1987). The iFat1 transgenic cassette was designed to simultaneously restore *Hprt* gene function, through introduction of human equivalents of the missing gene region, and insert the iFat1 transgene immediately upstream of this locus. Correctly targeted E14Tg2a clones were therefore positively selected based on resistance to hypoxanthine, aminopterin and thymidine (HAT) medium, which effectively blocks the de-novo pathway of nucleotide synthesis. Targeted transgenesis through restoration of Hprt function in E14Tg2a and E14Tg2a-derivatives is a commonly used approach for the generation of transgenic mouse lines (Bronson et al. 1996; Cvetkovic et al. 2000; Evans et al. 2000; Imrie et al. 2012).

The presence of successful recombination events in HAT resistant E14Tg2a clones was validated by southern blot analysis prior to generation of male chimeras through C57BL/6 J blastocyst injection. Highly chimeric males were bred with C57BL/6 J females to generate F1 progeny. Germ-line transmission of the iFat1 transgene to F1 progeny was confirmed by PCR and southern blot analysis. The resultant iFat1 F1 female heterozygous progeny were subsequently transferred from GenOway to the University of Guelph, at which point a breeding colony of heterozygous females was established. Since the iFat1 transgene is X-linked, males cannot inherit this transgene from paternal origins. The iFat1 breeding colony was established and is maintained through backcrossing of iFat1 heterozygous females with wildtype FC57BL/6 N males (Charles River). This strategy ensures that a subset of females and males within every generation inherits the iFat1 transgene.

### Plasmids

The DMA1-express plasmid (9771 bp), a conditional expression vector containing the iFat1 transgenic construct, was obtained from GenOway (Lyon, France). All other plasmids were obtained through the Addgene plasmid repository (www.addgene.org), deposited by Dr. Connie Cepko (Harvard Medical School). pCAG-Cre (5871 bp) is a plasmid in which the Cre expression is under direct control of the constitutively expressed CAG promoter (Addgene plasmid 13775) (Matsuda and Cepko 2007) The backbone vector of pCAG-Cre, pCAGEN (4798 bp), was used as a negative control (Addgene plasmid 11160) (Matsuda and Cepko 2004). All plasmids were purified using Wizard^®^ *Plus* Maxipreps DNA Purification System (Promega, A7270). Purified plasmid DNA was eluted in 1.5 mL DEPC treated water and quantified via spectrophotometry (ThermoScientific NanoDrop 2000).

### Cell Culture and Co-transfection

Functionality of the iFat1 transgene were screened in vitro via co-transfection experiments employing HEK 293T cells and the transfecting agent polyethylenimine (PEI) (Sigma Aldrich 408727), both of which were generously provided by Dr. Nina Jones (Department of Molecular and Cellular Biology, University of Guelph).

For routine maintenance, HEK 293T cells were grown in a standard culture medium composed of high-glucose DMEM (ThermoScientific SH30022.01), supplemented with 10 % FBS (Sigma F1051) and 1 % penicillin/streptomycin (Sigma A5955). For co-transfection experiments, cells were seeded at a density of approximately 0.5 × 10^6^ cells per 10 cm culture plate. The next day standard culture medium was aspirated and replaced with 10 mL of freshly prepared 100 μM linoleic acid (LA) supplemented DMEM containing 5 % FBS, 1 % penicillin/streptomycin and 0.1 % BSA (Roche 10735078001), as substrate, for a period of 72 h. Twenty-four hours following initiation of fatty acid treatment, cells were treated with either [a] transfecting agent alone in the absence of plasmid DNA [b] DMA1-express and pCAGEN (iFat1/Empty) or [c] DMA1-express and pCAG-Cre (iFat1/Cre). For each co-transfection reaction, 750 μL of OptiMEM serum free medium (Invitrogen Gibco^®^ 31985) and 25 μL of 2 mg/mL PEI were added to a total of 12 μg, of equally balanced, plasmid DNA. The transfection solution was briefly vortexed then incubated at room temperature for 5 min prior to edge-wise application to cell plates. 48 h post-transfection cells were pelleted by centrifugation at 500×*g* for 5 min at 4 °C. Supernatant was discarded and samples were subsequently rinsed twice in PBS by sequential re-suspension, centrifugation and supernatant disposal. Resulting cell pellets were directly processed for lipid extraction.

### Experimental animals

Male heterozygous Tam-Cre mice (Jackson Laboratories) were bred with female heterozygous iFat1 mice, which had been backcrossed onto a C57BL/6 background for 3 generations. Litters were weaned at 3 weeks of age, genotyped and male progeny identified as either: Tam-Cre^(−/−)^/iFat1^(−)^ (WT), Tam-Cre^(−/−)^/iFat1^(+)^ (iFat1) and Tam-Cre^(+/−)^/iFat1^(+)^ (Tam-Cre/iFat1) were used for the purpose of this study. Potential Cre-independent background expression characteristics of the iFat1 transgene were screened for using WT and iFat1 mice. For this, mice were maintained on the experimental diet throughout the study duration but received no additional treatment. Conversely, the Cre-inducible activation profile of the iFat1 transgene was considered using Tam-Cre/iFat1 double hybrid mice. Commencing at 6–7 weeks of age, Tam-Cre/iFat1 double hybrids were treated with either 100 μL of 10 mg/mL tamoxifen free-base (Sigma Aldrich, T-5648) suspended in corn-oil (Sigma C8267) or 100 μL of vehicle control, via one daily intraperitoneal (IP) injection administered for a course of five consecutive days.

Breeders and their progeny were maintained on a specially formulated modified AIN-93G diet containing 10 % w/w safflower oil diet (Research Diets, #D04092701), rich in LA. The fatty acid composition of this experimental diet has been reported elsewhere (MacLennan et al. 2013). Mice were housed 1–3/cage in a temperature and humidity controlled room on a diurnal 12 h light/dark cycle. In order to prevent potential cross-contamination by coprophagy between groups, mice were separately housed based on treatment strategy/group (Brake et al. 2004). All mice were euthanized at 9–10 weeks of age by CO_2_ asphyxiation. Kidney, liver, skeletal muscle and brain tissues were immediately harvested, snap frozen in liquid nitrogen and stored at −80 °C for later analysis. The animal utilization protocol for this study was approved by, and conducted in accordance with, the University of Guelph’s Animal Care Committee (protocol 12G002).

### Genotyping by PCR

Briefly, tail biopsies were incubated overnight, at 55 °C, with proteinase K (Invitrogen 25530-015) and tail lysis buffer (50 mM Tris, 100 mM NaCl, 1 % sodium dodecyl sulphate and 25 mM EDTA). DNA was extracted from digested tail samples with buffer saturated phenol (Invitrogen Ultrapure™ 15513-039), precipitated via ethanol washing and re-suspended in 30 μL of Tris–EDTA buffer. For each sample, 0.5 μL of genomic DNA was combined with 24.5 μL of PCR master-mix solution and amplified using an Applied Biosystems Veriti 96 Well Thermal Cycler. To screen samples for presence of the Tam-Cre transgene, PCR master mix was prepared to a final concentration of 0.2 mM dNTP (ThermoScientific Fermentas R0192), 2 mM MgCl_2_ and 0.625 units Platinum^®^ Taq DNA polymerase (Invitrogen 10966-034) and 0.5 μM each of specified primers (Ventura et al. 2007). Samples were incubated at 94 °C for 180 s, followed by 35 cycles of 94 °C for 30 s, 58 °C for 90 s and 72 °C for 60 s. Samples were held at a final extension temperature of 72 °C for 300 s. For iFat1 reactions, PCR master mix was composed of 0.3 mM dNTP, 10× PCR buffer, 1.5 mM MgCl_2_ and 1.5 units of Platinum ^®^ Taq and 0.4 μM each of: 5′-ACGTCAGTAGTCATAGGAACTGCGGTCG-3′(F) and 5′-CCAACCGGTGGGACATTT GAGTTG-3′(R). Samples were incubated at 94 °C for 120 s, followed by 35 cycles of 94 °C for 30 s, 55 °C for 30 s and 68 °C for 60 s. Samples were held at a final extension temperature of 68 °C for 420 s. This protocol amplifies a 325 bp sequence of the iFat1 transgene.

Amplified DNA was loaded on a 2 % agarose gel containing ethidium bromide (Fisher Scientific BP1302-10) and separated via gel electrophoresis. Gels were imaged under UV light using the FluorChem HD2 imaging system.

### Tamoxifen Preparation

A stock solution of 10 mg/mL tamoxifen free-base solubilized in ethanol/corn-oil was freshly prepared immediately before initiation of tamoxifen treatment and stored, protected from light, at 4 °C for the duration of the 5 day induction period. Briefly, 100 μL of anhydrous ethanol was added to a 10 mg aliquot of tamoxifen free-base and heated at 55 °C on dry heat-block with frequent intermittent vortexing to facilitate tamoxifen dissolution. 900 μL of sterile corn-oil was added and the solution was sonicated to homogeneity (30 s). Vehicle control was prepared in an analogous fashion, through omission of tamoxifen from the protocol.

### Lipid extraction

Total lipids were extracted from both cell culture and tissues using a modified method of Folch et al. (1957). For in vitro analysis, cell pellets were suspended in 1 mL of 0.1 M potassium chloride and immediately transferred into pre-chilled acid washed tubes. A 2:1 mixture of chloroform (Fisher Scientific C298-4): methanol (Fisher Scientific A452-4) was freshly prepared and 4 mL was added to each sample. Samples were vortexed for 1 min, flushed with a gentle stream of nitrogen gas and incubated at 4 °C overnight. Samples were centrifuged at 340×*g* for 10 min to separate phases. The lipid-containing chloroform layer was transferred into a disposable glass culture tube. Extracted lipids were dried under a gentle stream of nitrogen and reconstituted in 100 μL of chloroform for subsequent fatty acid analysis. For in vivo analysis, total lipids were extracted from 50 to 100 mg tissue sections. Briefly, tissue samples were homogenized on ice in 2.5 mL of 0.1 M potassium chloride (Fisher PowerGen 125) and subsequently prepared for lipid extraction using equivalent final solvent concentrations and extraction procedures as outlined above. Following centrifugation, the chloroform layer of each sample was transferred to a corresponding pre-weighed acid washed tube, dried under a gentle stream of nitrogen and reconstituted at a concentration of 10 mg/mL in chloroform.

### Fatty acid analysis

Phosphatidylethanolamine (PE), phosphatidylcholine (PC), phosphatidylserine (PS) and phosphatidylinositol (PI) fractions were isolated from total lipid extracts by thin layer chromatography using silica H-plates (VWR 5721-7) as previously outlined (5). Fatty acid methyl esters were prepared by incubating in 2 mL of hexane (EMD HX0295-1) and 2 mL 14 % boron trifluoride-methanol solution (Sigma Aldrich B1252) at 100 °C for 90 min. 2 mL of double deionized water was added to each tube to stop the methylation reaction. Samples were vortexed and phases were separated via centrifugation at 340×*g* for 10 min. The upper hexane layer was transferred to a 2 mL glass vial (Agilent 5182-0714), dried under a gentle stream of nitrogen gas and reconstituted in hexane for analysis by gas–liquid chromatography.

Fatty acid methyl ester composition of phospholipid fractions was resolved using an Agilent 7890A gas chromatograph (Agilent Technologies, Santa Clara USA) equipped with a flame ionization detector and a fused-silica polyethylene glycol capillary column (DB-FFAP; 15 m, 0.1 mm internal diameter, 0.1 μm film thickness, Agilent Technologies 127-32H2). Samples were injected in split mode (1:200) using hydrogen as the carrier gas (30 mL/min) and eluted according to the following temperature program: 150 °C for 20 s, ramp at 35 °C/min, hold at 170 °C for 3 min, ramp at 9 °C/min, hold at 225 °C for 30 s, ramp at 80 °C/min, hold at 245 °C for 2.2 min. Chromatograms were generated using the Agilent EZChrom Elite GC Data System (version 3.3.2). Individual fatty acid peak identities were verified by comparison with peak retention times of known fatty acid standards (Nu-Check Prep, Elysian, MN), and expressed as relative percent composition of total fatty acids.

### Statistical analysis

All fatty acid data is expressed as mean ± standard deviation (SD). Statistical analysis was conducted using SAS v9.1. ANOVA with Tukey post hoc test was used to analyze fatty acid data from in vitro experiments. Where appropriate, data was logarithmically transformed to produce normality. Fatty acid data from in vivo experiments were analyzed by using Student’s *t* tests. Significance was set at *p* < 0.05 for all analyses.

## Results

### The iFat1 transgene permits conditional n-3 PUFA enrichment in vitro

**Fig. 3:**
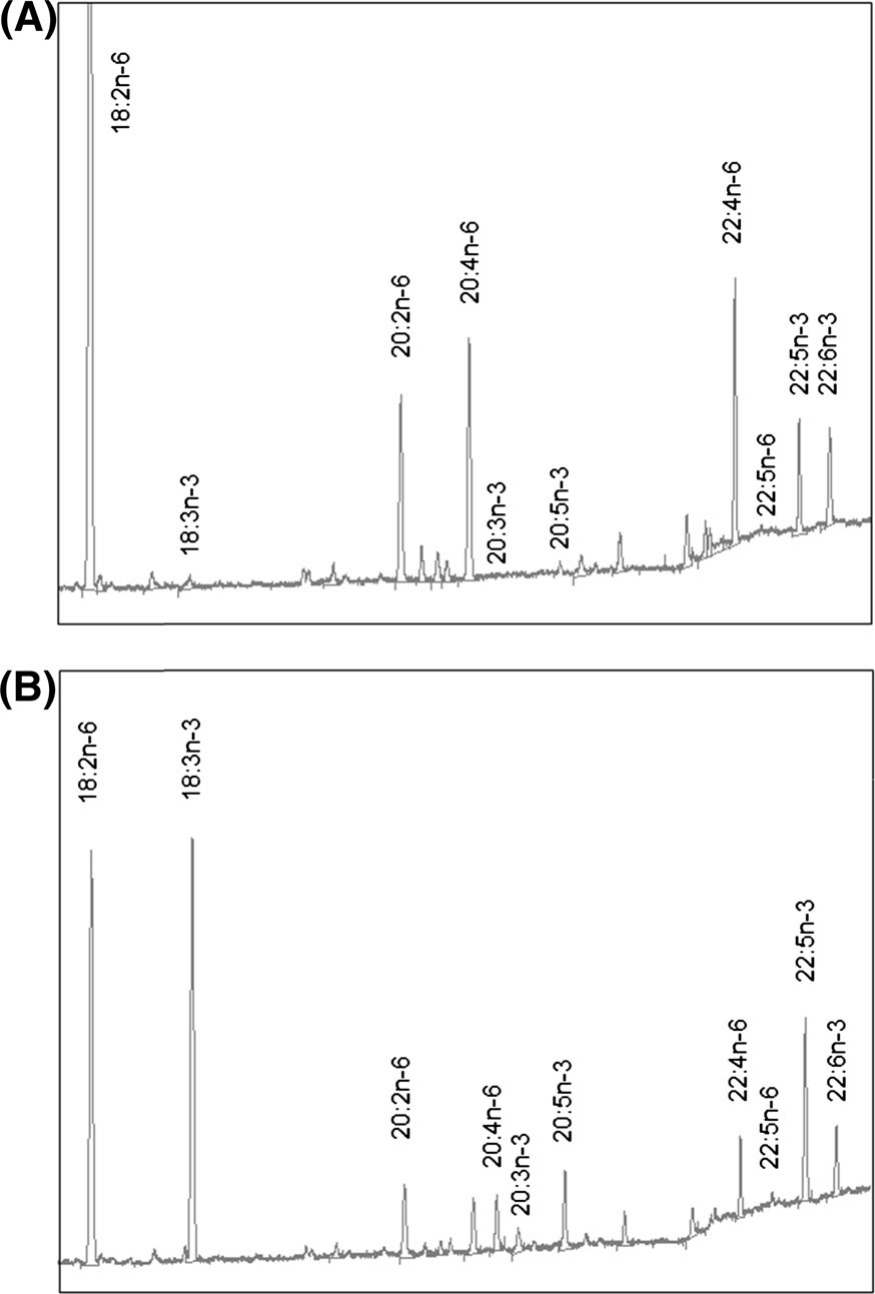
Representative gas chromatography trace of PE fraction of co-transfected HEK 293T cells. In relation to cells co-transfected with (**a**) iFat1/Empty, co-transfection with (**b**) iFat-1/Cre resulted in marked elevations in n-3 PUFA and corresponding reductions in n-6 PUFA species. For detailed PUFA compositional changes within phospholipid fractions refer to Table 1

**Fig. 4:**
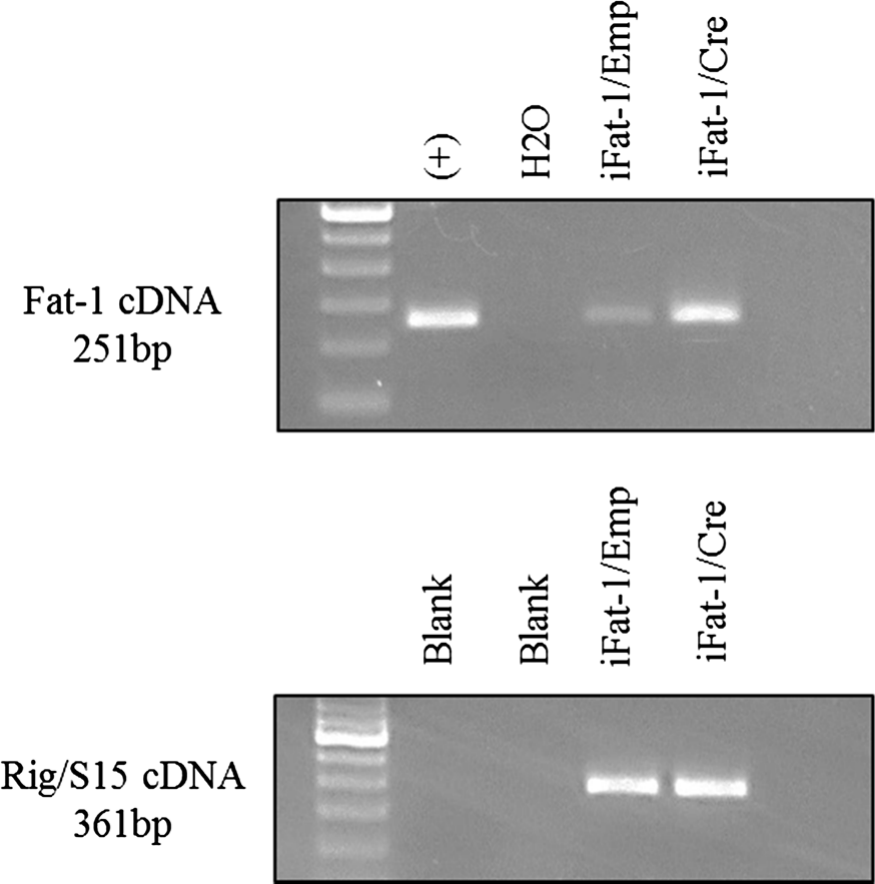
Transcriptional expression characteristics of the iFat-1 transgene in vitro. Cells were co-transfected with iFat-1/Emp or iFat-1/Cre and harvested for total cellular RNA 24 h post-transfection. RT-PCR was performed to probe for the presence of transcripts corresponding to the fat-1 coding sequence. Hepatic cDNA obtained from total hepatic RNA of a constitutive fat-1 mouse was included as a reference (+) control. RT-PCR for rig/S15 cDNA, a constitutively expressed housekeeping gene encoding a small ribosomal subunit protein, was included as an indirect marker of RNA quality for each sample as per manufacturer instructions (RNAqueous^®^- 4PCR kit). Results demonstrate the presence of Cre-independent expression from the iFat-1 transgene

In comparison to controls, iFat1/Cre co-transfected cells were observed to have pronounced alterations in n-3 and n-6 PUFA composition within membrane phospholipids. PUFA were selectively and differentially enriched in phospholipids. The PUFA compositional changes in HEK 293T cells were most marked within the PE and PC fractions. A representative chromatographic trace from the PE fraction of co-transfected cells is shown in Fig. 3 and details on the compositional changes of n-6 and n-3 PUFA species within the PE and PC fractions are reported in Table 1. Within the PE and PC fractions, α-linolenic acid (ALA) (*p* < 0.01) and LA (*p* < 0.01) were most notably enriched and reduced, respectively. However, significant increases in several downstream long-chain n-3 PUFA species, including EPA (*p* < 0.05) and 22:5n-3 (*p* < 0.01), with concomitant decreases in corresponding n-6 PUFA species including 20:4n-6 and 22:4n-6 (*p* < 0.01) were also evident. Collectively, the observed changes in fatty acid composition within the PE fraction was consistent with a balancing of the n-6/n-3 PUFA ratio (1.0, *p* < 0.01) in iFat1/Cre treated cells relative to controls. Results from other phospholipid fractions were in general agreement with those of the PE and PC fractions. Total saturated fatty acids (SFA), monounsaturated fatty acids (MUFA) and PUFA did not differ between experimental groups for any phospholipid fraction. It was observed that background Cre-independent mRNA expression from the iFat1 transgene was detected by RT-PCR (Fig. 4), however this did not correspond to changes in fatty acid composition. Total n-6 PUFA content (PE, PC) and total n-3 PUFA content (PE, PC) did not significantly differ between cells co-transfected with iFat1/Empty and control cells which had been treated with transfecting reagent alone (No DNA). Additionally, iFat1/Empty and transfection controls were demonstrated to possess comparable fatty acid composition for all individual PUFA species measured. Thus, these findings indicate that while the loxP flanked STOP cassette of the iFat1 transgene may not terminate transcription with 100 % efficiency, its translation block mechanism is sufficient to render PUFA phenotype strictly reliant on Cre as an activating factor.

**Table 1:** Fatty acid composition (% total) of phosphatidylethanolamine (PE) and phosphatidylcholine (PC) in co-transfected HEK 293T cells

Fatty acid	PE			PC		
	No DNA	iFat1/Empty	iFat1/Cre	No DNA	iFat1/Empty	iFat1/Cre
18:2n-6	29.1 ± 2.7^a^	31.4 ± 2.4^a^	16.3 ± 2.0^b^	34.4 ± 2.2^a^	32.6 ± 3.9^a^	13.9 ± 2.1^b^
20:4n-6	4.8 ± 0.9^a^	5.4 ± 0.4^a^	1.9 ± 0.2^b^	0.7 ± 0.1^a^	0.6 ± 0.0^a^	trace^b^
22:4n-6	3.3 ± 0.6^a^	4.5 ± 0.6^a^	2.0 ± 0.3^b^	0.3 ± 0.3	0.4 ± 0.1	0.2 ± 0.3
22:5n-6	n.d.	n.d.	n.d.	n.d.	n.d.	n.d.
18:3n-3	n.d.^b^	0.2 ± 0.4^b^	15.0 ± 0.5^a^	n.d.^c^	0.2 ± 0.1^b^	18.7 ± 0.6^a^
20:5n-3	n.d.^b^	n.d.^b^	2.6 ± 0.3^a^	n.d.^b^	n.d.^b^	0.5 ± 0.0^a^
22:5n-3	1.8 ± 0.1^b^	2.2 ± 0.3^b^	4.5 ± 0.5^a^	0.4 ± 0.2	0.5 ± 0.2	0.7 ± 0.0
22:6n-3	1.9 ± 0.6	1.9 ± 0.3	1.8 ± 0.3	trace	0.4 ± 0.1	0.4 ± 0.0
Total n-6	43.9 ± 4.5^a^	48.3 ± 2.0^a^	25.0 ± 2.3^b^	40.0 ± 2.1^a^	37.9 ± 4.2^a^	16.7 ± 2.5^b^
Total n-3	3.7 ± 0.5^b^	4.3 ± 0.9^b^	26.0 ± 0.5^a^	0.5 ± 0.1^b^	1.1 ± 0.3^b^	22.2 ± 0.6^a^
n-6/n-3	11.8 ± 0.6^a^	11.7 ± 2.9^a^	1.0 ± 0.1^b^	83.9 ± 15.1^a^	37.5 ± 14.5^b^	0.8 ± 0.1^c^
Total SFA	38.7 ± 8.7	33.1 ± 1.3	33.6 ± 2.1	47.0 ± 1.1	49.7 ± 3.9	48.7 ± 1.4
Total MUFA	13.5 ± 3.7	13.8 ± 0.3	15.1 ± 1.6	12.4 ± 3.0	10.9 ± 0.4	12.1 ± 1.7
Total PUFA	47.8 ± 5.1	53.1 ± 1.2	51.3 ± 2.4	40.6 ± 2.2	39.4 ± 4.0	39.2 ± 3.1

Cells were co-transfected with iFat1/Empty or iFat1/Cre. Cells treated with transfecting reagent in the absence of plasmid DNA (No DNA) were also included as controls for potential background difference in fatty acid composition attributable to Cre-independent expression of the iFat1 transgene. For each phospholipid fraction, means possessing different superscripts within rows denote significant differences between groups (*p* < 0.05)

### The iFat1 Transgene permits conditional n-3 PUFA enrichment in vivo

Analogous to the in vitro results, background Cre-independent mRNA expression of *fat*-*1* associated transcripts was detected within the hepatic tissue of mice carrying the iFat1 transgene (data not shown). However, the PUFA phenotype of liver tissue from WT and iFat1 (non treated) transgenic mice was similar (Table 2). The PUFA composition of kidney, muscle and brain phospholipids between WT and iFat1 transgenic mice were also closely matched (data not shown).

**Table 2:** Non-induced iFat1 mouse hepatic fatty acid composition

Fatty acid	PE		PC	
	WT	iFat1	WT	iFat1
18:2n-6	8.2 ± 0.7	6.6 ± 0.9	16.8 ± 1.5	19.3 ± 1.7
20:4n-6	30.7 ± 0.8	30.2 ± 0.7	n.d.	n.d.
22:4n-6	1.3 ± 0.1	1.4 ± 0.2	20.8 ± 2.5	22.7 ± 1.0
22:5n-6	7.5 ± 1.0	8.0 ± 1.3	n.d.	n.d.
18:3n-3	n.d.	n.d.	0.5 ± 0.1	0.6 ± 0.1
20:5n-3	n.d.	n.d.	4.3 ± 0.7	4.2 ± 0.7
22:5n-3	0.2 ± 0.1	trace	trace	trace
22:6n-3	2.2 ± 0.5	1.8 ± 0.1	0.9 ± 0.1	1.1 ± 0.2
Sum of n-6	49.6 ± 0.7	47.9 ± 1.1	49.6 ± 0.8*	45.7 ± 2.2
Sum of n-3	2.4 ± 0.5	2.0 ± 0.2	1.2 ± 0.2	1.0 ± 0.2
n-6/n-3	21.8 ± 5.7	24.8 ± 3.0	44.4 ± 10.7	47.5 ± 8.8
Total SFA	37.6 ± 0.8	38.9 ± 2.1	40.7 ± 0.6	42.1 ± 1.9
Total MUFA	10.4 ± 0.6	11.3 ± 1.4	8.5 ± 0.3	11.2 ± 2.2
Total PUFA	52.0 ± 0.5*	49.9 ± 1.1	50.7 ± 0.8*	46.7 ± 2.3

Phospholipid composition of select n-6 and n-3 PUFA in liver tissue from WT (n = 3/group) and iFat1 (n = 3/group): PE and PC. Results expressed as mean ± SD. For each phospholipid fraction, means possessing asterisk (*) within rows indicate significant differences between groups

**Fig. 5:**
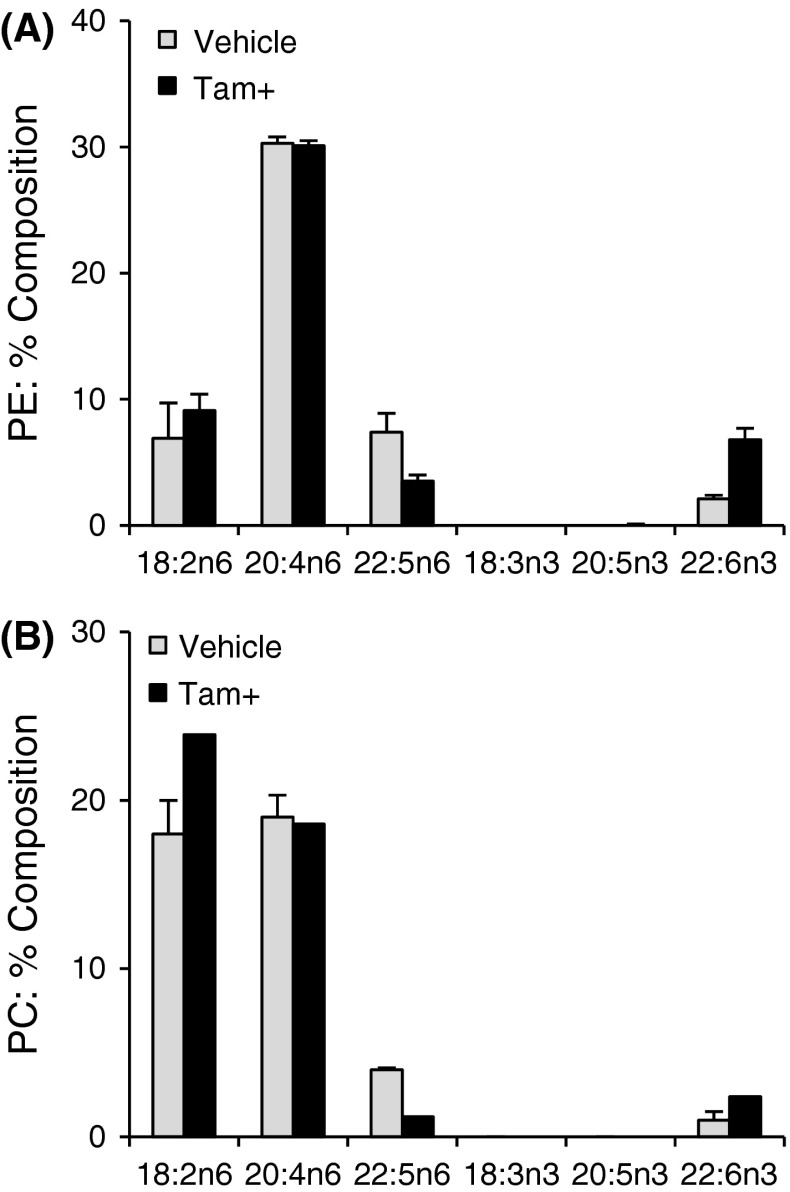
Relative fatty acid composition of major n-6 and n-3 PUFA species in the (A) PE and (B) PC fraction of liver tissue from Tam-Cre/iFat1 mice treated with vehicle or tamoxifen (Tam +). Results expressed as mean ± SD (n = 3/group). *Asterisk* denotes significant difference (*p* < 0.05) between groups by Student’s *t* test

Conditional expression characteristics of the iFat1 transgene were assessed using tamoxifen treated and vehicle treated Tam-Cre/iFat1 transgenic mice. The composition of major n-6 and n-3 PUFA within the PE and PC fraction of liver tissue are presented in Fig. 5 and detailed PUFA compositional data from each of these major phospholipid fractions are provided in Tables 3 and 4 respectively. In relation to vehicle treated controls, tamoxifen treatment of Tam-Cre/iFat1 mice resulted in relative enrichment in phospholipid total n-3 PUFA in each of the liver (PE, PC; *p* < 0.01), kidney (PE and PC; *p* < 0.01) and muscle (PE; *p* < 0.01, PC; *p* < 0.05). Within all of these tissue phospholipid fractions, DHA was the predominant n-3 PUFA species enriched. However lesser contributions to total relative n-3 PUFA enrichment from 22:5n-3 within the liver (PE and PC; *p* < 0.05), kidney (PC; *p* < 0.01, PE; *p* ≤ 0.05) and muscle (PE and PC; *p* < 0.01) were also observed. Moreover, relative reductions within the corresponding n-6 PUFA species, 22:5n-6 and 22:4n-6 were also observed within select phospholipid fractions from the liver and kidney as well as a small but significant overall decrease in total n-6 PUFA content in kidney and muscle tissue. Collectively, these PUFA compositional changes resulted in an approximate twofold reduction, or more, of the n-6/n-3 PUFA ratio in each of the liver (PE: −3.6; *p* < 0.01, PC: −2.3), kidney (PE: −2.4 and PC: −2.4; *p* < 0.01) and muscle (PE: −2.0 and PC: −1.9; *p* < 0.05) tissues. However, PUFA compositional changes within the brain tissue were less discernible, with only a small but significant difference in the n-6/n-3 PUFA ratio evident within the PC fraction (−1.3 fold; *p* < 0.05), which was neither associated with a marked difference in total n-6 PUFA or total n-3 PUFA between tamoxifen and vehicle treated mice. Total SFA, MUFA and PUFA did not generally differ between experimental groups.

**Table 3:** Cre-inducible iFat1 expression characteristics in vivo: PE fraction

FA	Liver		Kidney		Muscle		Brain	
	Vehicle	Tam+	Vehicle	Tam+	Vehicle	Tam+	Vehicle	Tam+
18:2n6	6.9 ± 2.8	9.1 ± 1.3	4.9 ± 1.0	5.0 ± 0.5	7.5 ± 0.4	7.5 ± 0.6	0.4 ± 0.0	0.4 ± 0.1
18:3n6	trace	trace	n.d.	n.d.	n.d.	n.d.	n.d.	n.d.
20:2n6	0.5 ± 0.1	0.5 ± 0.1	0.4 ± 0.1	0.4 ± 0.1	0.4 ± 0.1	0.5 ± 0.1	0.3 ± 0.0	0.3 ± 0.1
20:3n6	1.0 ± 0.1	0.8 ± 0.0	0.6 ± 0.0	0.5 ± 0.1	0.7 ± 0.0	0.6 ± 0.1	0.5 ± 0.0	0.4 ± 0.0
20:4n6	30.3 ± 0.5	30.1 ± 0.4	32.9 ± 1.2	34.8 ± 2.0	13.6 ± 1.5	13.1 ± 0.7	15.3 ± 0.6	16.7 ± 0.2*
22:2n6	trace	n.d.	n.d.	n.d.	n.d.	n.d.	n.d.	trace
22:4n6	1.3 ± 0.1*	1.1 ± 0.1	2.2 ± 0.3	1.8 ± 0.1	6.3 ± 0.3	6.9 ± 0.2	6.8 ± 0.1	6.9 ± 0.2
22:5n6	7.4 ± 1.5*	3.5 ± 0.5	11.2 ± 1.6*	6.0 ± 1.2	25.5 ± 2.4	22.1 ± 2.9	17.0 ± 0.3	15.2 ± 2.3
18:3n3	n.d.	n.d.	n.d.	n.d.	trace	n.d.	trace	n.d.
18:4n3	n.d.	n.d.	n.d.	n.d.	n.d.	n.d.	n.d.	n.d.
20:3n3	n.d.	n.d.	n.d.	n.d.	n.d.	n.d.	n.d.	n.d.
20:5n3	n.d.	n.d.	n.d.	n.d.	n.d.	0.5 ± 0.2	n.d.	n.d.
22:3n3	n.d.	n.d.	n.d.	n.d.	n.d.	n.d.	n.d.	n.d.
22:5n3	trace	0.4 ± 0.1*	0.2 ± 0.0	0.7 ± 0.2	0.6 ± 0.1	1.4 ± 0.2*	0.2 ± 0.0	0.3 ± 0.0
22:6n3	2.1 ± 0.3	6.8 ± 0.9*	3.5 ± 0.2	7.8 ± 1.5*	2.8 ± 0.3	4.6 ± 0.5*	10.0 ± 0.9	13.2 ± 3.6
Total n-6 PUFA	47.7 ± 0.9	45.2 ± 1.8	52.2 ± 0.4*	48.5 ± 0.8	53.9 ± 0.7*	50.7 ± 1.6	40.2 ± 0.6	40.0 ± 2.3
Total n-3 PUFA	2.2 ± 0.4	7.3 ± 1.0*	3.7 ± 0.2	8.5 ± 1.4*	3.5 ± 0.4	6.4 ± 0.9*	10.3 ± 0.9	13.5 ± 3.5
n-6/n-3 PUFA	22.2 ± 4.6*	6.3 ± 1.1	14.0 ± 1.0*	5.8 ± 0.8	15.6 ± 2.0*	8.0 ± 1.4	3.9 ± 0.3	3.1 ± 0.9
Total SFA	40.0 ± 1.3	39.3 ± 1.3	33.1 ± 0.2	32.2 ± 1.2	35.1 ± 0.9	35.6 ± 0.5	33.6 ± 1.4	32.9 ± 2.1
Total MUFA	10.2 ± 0.7*	8.3 ± 0.4	10.9 ± 0.2	10.7 ± 1.0	7.4 ± 1.0	7.2 ± 0.5	15.9 ± 1.8	13.5 ± 1.3
Total PUFA	49.9 ± 0.8	52.4 ± 1.4	56.0 ± 0.2	57.0 ± 2.1	57.5 ± 0.6	57.1 ± 1.0	50.5 ± 1.3	53.6 ± 1.3*

Relative PUFA composition within PE fraction of tissues from Tam-Cre/iFat1 mice treated with corn-oil (Vehicle) or tamoxifen (Tam +). Results expressed as mean ± SD (n = 3/group). Asterisk (*) denotes significant difference (*p* < 0.05) between groups by Student’s *t* test

**Table 4:** Cre-inducible iFat1 expression characteristics in vivo: PC fraction

FA	Liver		Kidney		Muscle		Brain	
	Vehicle	Tam+	Vehicle	Tam+	Vehicle	Tam+	Vehicle	Tam+
18:2n6	18.0 ± 6.0	23.9 ± 2.0	12.1 ± 2.6	13.2 ± 1.2	17.7 ± 0.9	19.1 ± 0.5	0.9 ± 0.1	0.9 ± 0.1
18:3n6	0.4 ± 0.1	0.3 ± 0.1	0.2 ± 0.0	trace	trace	trace	n.d.	n.d.
20:2n6	0.5 ± 0.1	0.5 ± 0.1	0.7 ± 0.1	0.8 ± 0.1	0.5 ± 0.1	0.6 ± 0.0	0.3 ± 0.1	0.4 ± 0.0
20:3n6	2.4 ± 0.1*	1.6 ± 0.2	1.3 ± 0.1	1.1 ± 0.2	1.6 ± 0.0*	1.3 ± 0.1	0.2 ± 0.1	0.3 ± 0.0
20:4n6	19.0 ± 4.5	18.6 ± 1.3	14.9 ± 2.1	14.8 ± 1.8	18.8 ± 0.5	18.8 ± 0.9	6.0 ± 0.2	6.5 ± 0.4
22:2n6	n.d.	n.d.	n.d.	n.d.	n.d.	n.d.	n.d.	n.d.
22:4n6	0.5 ± 0.1	0.3 ± 0.1	1.5 ± 0.1*	1.0 ± 0.1	1.9 ± 0.3	1.9 ± 0.2	0.9 ± 0.0*	0.8 ± 0.1
22:5n6	4.0 ± 1.1*	1.2 ± 0.3	12.2 ± 2.0*	5.7 ± 1.7	4.8 ± 1.2	3.4 ± 0.6	2.7 ± 0.1*	2.2 ± 0.3
18:3n3	n.d.	n.d.	n.d.	n.d.	trace	trace	n.d.	n.d.
18:4n3	n.d.	n.d.	n.d.	n.d.	n.d.	n.d.	n.d.	n.d.
20:3n3	n.d.	n.d.	n.d.	n.d.	n.d.	n.d.	n.d.	n.d.
20:5n3	n.d.	n.d.	n.d.	trace	n.d.	0.3 ± 0.2	n.d.	n.d.
22:3n3	n.d.	n.d.	n.d.	n.d.	n.d.	n.d.	n.d.	n.d.
22:5n3	n.d.	0.2 ± 0.0*	0.5 ± 0.1	0.9 ± 0.1*	0.2 ± 0.0	0.5 ± 0.1*	n.d.	trace
22:6n3	1.0 ± 0.1	2.4 ± 0.5*	4.5 ± 0.4	9.7 ± 2.1*	0.7 ± 0.0	1.0 ± 0.0*	1.3 ± 0.0	1.7 ± 0.4
Total n-6 PUFA	44.8 ± 1.9	46.5 ± 1.4	42.7 ± 1.3*	36.6 ± 1.3	45.5 ± 2.1	45.2 ± 1.4	11.0 ± 0.4	11.0 ± 0.1
Total n-3 PUFA	1.1 ± 0.2	2.6 ± 0.5*	5.0 ± 0.4	10.6 ± 2.1*	1.0 ± 0.0	2.0 ± 0.3*	1.3 ± 0.0	1.7 ± 0.3
n-6/n-3 PUFA	42.4 ± 5.5*	18.3 ± 2.7	8.6 ± 0.6*	3.6 ± 0.7	44. ± 0.8*	23.4 ± 4.2	8.7 ± 0.4*	6.5 ± 1.2
Total SFA	42.9 ± 0.4	43.7 ± 1.5	44.0 ± 0.4	45.3 ± 1.6	42.9 ± 0.7	43.8 ± 0.7	60.6 ± 0.3	60.5 ± 0.4
Total MUFA	11.3 ± 1.7*	7.2 ± 0.5	8.3 ± 1.1	7.5 ± 0.6	10.6 ± 1.5	9.0 ± 0.7	27.2 ± 0.5	26.7 ± 0.5
Total PUFA	45.9 ± 2.0	49.1 ± 1.7	47.7 ± 1.5	47.2 ± 2.2	46.5 ± 2.1	47.1 ± 1.2	12.2 ± 0.4	12.8 ± 0.4

Relative PUFA composition within PC fraction of tissues from Tam-Cre/iFat1 mice treated with vehicle (Sham) or tamoxifen (Tam +). Results expressed as mean ± SD (n = 3/group). Asterisk (*) denotes significant difference (*p* < 0.05) between groups by Student’s *t* test

## Discussion

While research employing the fat-1 transgenic mouse has lent strong support to the protective health benefits of lifelong n-3 PUFA exposure, the constitutive nature of transgene expression precludes the application of this model for investigating issues of timing of n-3 PUFA exposure. The iFat1 transgenic mouse, which carries a Cre-inducible version of the *C. elegans* *fat*-*1* gene has been developed as a potential tool to address this research gap. In contrast to the original fat-1 transgene described by Kang et al. (2004), the iFat1 regulatory unit has been adapted to contain a floxed STOP cassette, thus introducing an overarching mechanism through which the timing and/or pattern of endogenous n-3 PUFA enrichment can be differentially controlled. This study describes the novel iFat1 model containing a transgene capable of facilitating Cre-inducible synthesis of endogenous n-3 PUFA enrichment within mammalian cells and tissues.

The capacity of mammalian cells to endogenously convert n-6 PUFA to n-3 PUFA through heterologous expression of the humanized *C. elegans* *fat*-*1* gene was first reported by Kang et al. (2001). In this early report, an adenovirus mediated approach was used to specifically introduce the *fat*-*1* gene into cultured rat cardiac myocytes, the net result of which was a balanced n-6/n-3 PUFA ratio in total cellular lipids within a 48 h period; an ~15 fold reduction from that of controls (Kang et al. 2001). This balancing effect has been subsequently paralleled in various other mammalian cell-lines of both murine (An et al. 2012; Ge et al. 2002b) and human origin (Ge et al. 2002a; Xia et al. 2005). Within the present study, simultaneous introduction of iFat1 and Cre into HEK 293T cells was associated with a complete balancing of the n-6/n-3 PUFA ratio of membrane phospholipids. This balancing effect was directly attributable to an increase in the relative proportion of n-3 PUFA at the expense of corresponding n-6 PUFA species evident within all major phospholipid fractions, though most pronounced within PE (*p* < 0.01) and PC (*p* < 0.01) phospholipids. Surprisingly, DHA was not enriched. However, it is likely that additional incubation time was needed to allow for further elongation and desaturation from ALA given that downstream fatty acids were not enriched to the same extent as ALA, which is a direct product of desaturation of LA by the FAT-1 protein. Collectively, these results indicate that the iFat1 transgene is capable of producing Cre-inducible regulation over endogenous n-3 PUFA enrichment within mammalian cells in a manner that is generally consistent with the original *fat*-*1* construct.

In extension to the in vitro study arm which validated Cre-inducibility, the next phase of work was to demonstrate temporal inducibility and functionality of the model in an in vivo system. Tamoxifen dependent recombinase systems, such as the Tam-Cre mouse model is used widely, thus selected to validate the in vivo utility of the iFat1 transgene. Indeed, within 3 weeks of iFat1 transgene induction the phospholipid n-6/n-3 PUFA ratio of liver, kidney and muscle was observed to be approximately twofold lower than that of corresponding tissues taken from vehicle treated controls. While the observed percent composition of n-3 PUFA enrichment within the Cre-induced Tam-Cre/iFat-1 mice appears lower than that previously reported with the constitutive fat-1 model (Boudrault et al. 2010; Kang et al. 2004; Smith et al. 2010; Sun et al. 2011) and further substantiated within our lab using age-matched fat-1 reference controls (data not shown), it is not unexpected given that tissues were examined 3 weeks post transgene induction, as compared to constitutive expression. Interestingly, despite clear evidence supporting the ability of the iFat1 mouse model to facilitate Cre-inducible n-3 PUFA enrichment within peripheral tissues, brain PUFA composition was not found to markedly differ between tamoxifen treated Tam-Cre/iFat1 mice and vehicle treated controls. However, a similar lack of brain phenotype has also been previously reported in combination with the Tam-Cre model employed within the present study as well as other ROSA26 promoter driven tamoxifen inducible Cre lines. It is speculated to be the function of reduced tamoxifen tissues access arising from the blood–brain-barrier and/or low ROSA26 driven Cre expression within brain tissue (Hameyer et al. 2007; Ripoche et al. 2013; Seibler et al. 2003). Further refinement of the Tamoxifen induction in terms of dosage, duration and timing of exposure may also yield further enhancements in tissue n-3 PUFA.

In conclusion, this study provides in vitro and in vivo evidence that mammalian cells and tissues can be modified through incorporation of the iFat1 transgene to endogenously convert n-6 to n-3 PUFA in a manner which is reliant on Cre as an activating factor. These models have potential application as versatile tools in addressing the temporal and/or tissue specific effects of n-3 PUFA in disease prevention and treatment when combined with other Cre expressing models.

## Abbreviations

HEK: Human embryonic kidney
EPA: Eicosapentaenoic acid
DHA: Docosahexaenoic acid
*C. elegans*: *Caenorhabditis elegans*
Hprt: Hypoxanthine phosphoribosyltransferase
HAT: Hypoxanthine, aminopterin and thymidine
PEI: Polyethylenimine
LA: Linoleic acid
IP: Intraperitoneal
PE: Phosphatidylethanolamine
PC: Phosphatidylcholine
PS: Phosphatidylserine
PI: Phosphatidylinositol
ALA: α-Linolenic acid
SFA: Saturated fatty acid
MUFA: Monounsaturated fatty acid
4-OHT: 4-Hydroxytamoxifen
